# The correlates of diagnostic instability in eating disorders: the role of psychopathology, environmental risk factors, personality and genes

**DOI:** 10.1186/2050-2974-1-S1-O43

**Published:** 2013-11-14

**Authors:** Isabel Krug, Matthew Fuller-Tyszkiewicz, Nadia Micali, Marija Anderluh, Fernando Fernandez-Aranda, Kate Tchanturia, Andreas Karwautz, Gudrun Wagner, David Collier, Janet Treasure

**Affiliations:** 1 Melbourne University, Australia; 2 Deakin Universiy, Australia; 3 University College London, UK; 4 University Medical Centre Ljubliana, Slovenia; 5 University Hospital of Bellvitge, University of Barcelona, Spain; 6 King's College London, UK; 7 Medical University of Vienna, Austria

## Aim

To assess the occurrence of diagnostic cross-over in Eating Disorders (EDs) and assess its relationship with psychopathology, environmental risk factors, personality and genes.

## Method

Participants were 316 ED patients. The EATATE part 1 (a semi-structured interview) was used to examine diagnostic cross-over in EDs. The Eating Disorder Inventory (EDI-2), Temperament and Character Inventory (TCI-R), Oxford Risk Factor Interview (ORFI), the EATATE part 2 [used to assess obsessive-compulsive personality (OCPD) traits and impulsive behaviours] and four candidate genes (5-HT2A, BDNF, 5-HTTLPR, DRD4) were used to assess differences in cross-over patterns.

## Results

The majority of ED patients (65%) presented with diagnostic instability. The most commom cross-over change (23.42%) was from Anorexia Nervosa Restrictive (AN-R) subtype to a bulimic disorder. Significant differences across four ED cross-over groups [1.) AN-R to bulimic disorder; 2.) bulimic disorder to AN-R (5.6%); 3.) threshold ED to EDNOS (10.76%); 4.) EDNOS to threshold ED (6.7%)], a stable group (34.5%) and a remitted group (18.67%) were obtained the EDI bulimia, asceticism and impulse regulation subscales, the TCI-R self-directideness and cooperativeness subscales, childhood OCPD traits and impulsive behaviours (*p*<.05). No significant associations were found for environmental risk factors, the four candidate genes and diagnostic cross-over.

## Conclusions

The findngs of the current study indicate that diagnostic instability is very common in EDs and that especially psychopathological and personality correlates should be taken into consideration when treating patients with cross-over patterns.

This abstract was presented in the **Anorexia Nervosa – Characteristics and Treatment** stream of the 2013 ANZAED Conference.

